# *In Vivo* and *In Vitro* Anti-Tumor Effects of Fungal Extracts

**DOI:** 10.3390/molecules19022546

**Published:** 2014-02-21

**Authors:** Hung-Tsung Wu, Feng-Hwa Lu, Yu-Chu Su, Horng-Yih Ou, Hao-Chang Hung, Jin-Shang Wu, Yi-Ching Yang, Chih-Jen Chang

**Affiliations:** 1 Research Center of Herbal Medicine, New Drugs and Nutritional Supplements, Research and Services Headquarters, National Cheng Kung University, Tainan 70403, Taiwan; E-Mail: microbe0905702@yahoo.com.tw; 2 Department of Family Medicine, National Cheng Kung University Medical College and Hospital, Tainan 70403, Taiwan; E-Mails: fhlu@mail.ncku.edu.tw (F.-H.L.); jins@mail.ncku.edu.tw (J.-S.W.); yiching@mail.ncku.edu.tw (Y.-C.Y.); 3 Institute of Basic Medical Sciences, College of Medicine, National Cheng Kung University, Tainan 70403, Taiwan; E-Mail: horseofblackchu@gmail.com; 4 Department of Internal Medicine, National Cheng Kung University Medical College and Hospital, Tainan 70403, Taiwan; E-Mails: wahoryi@mail.ncku.edu.tw (H.-Y.O.); haochang.hung@gmail.com (H.-C.H.)

**Keywords:** apoptosis, cancer, *F. pinicola*, sarcoma-180 cells, tumors

## Abstract

Fungal extracts are extensively used as nutritional supplements in Far-Eastern Asia. In this study, we aimed to evaluate the anti-cancer activities of some different fungal species against different cancer cell lines. The water or ethanol extracts of *Fomitopsis pinicola* (*F. pinicola*), *Ganoderma sinense*, *Fomitopsis officinalis*, *Polyporus melanopus*, and *Taiwanofungus camphorates* were used to evaluate the anti-cancer activities in various cancer cells. We found that all of the fungi ethanol extracts used in this study exert anti-cancer activities* in vitro*, whereas water extracts show lower inhibitory activities as determined by 3-(4,5-methylthiazol-2-yl)-2,5-diphenyltetrazolium bromide assays. Among the tested fungi species, *F. pinicola* ethanol extract exerts the most significant anti-cancer activity (growth inhibitory ratio 82.8%, *p* < 0.001) by increasing cell apoptosis. Moreover, *F. pinicola* ethanol extract significantly decreased tumor size (tumor growth inhibitory ratio 54%, *p* < 0.05) and increased the lifespan in mice bearing sarcoma-180 tumors. Taken together, this is the first study indicating the anti-tumor effect of *F. pinicola in vivo* and* in vitro*. *F. pinicola* ethanol extract induces cell apoptosis to exert a significant anti-tumor activity, with potential to be a new alternative anti-tumor medicine.

## 1. Introduction

Fungi species, including reishi mushrooms are attractive sources of physiologically functional foods and drug precursors, displaying a wide range of pharmacological activities such as anticancer, anti-inflammation and immunomodulatory effects [1]. A wide range of fungal species are extensively used as nutritional supplements in Asia and are being increasingly used in Western countries too in conjunction with or in place of allopathic medicines [2]. Fungal extracts produce a variety of biologically active compounds and can therefore exert multiple biological functions. Increased evidence indicates that fungal extract consumption may protect against certain types of cancers, specifically gastrointestinal and breast cancer [3,4,5]. However, the efficacy of different fungal species in curing cancer and as preventive treatments remains as questionable and has not been scientifically evaluated.

*Fomitopsis pinicola* (*F. pinicola*) is a brown-rot fungus, that grows on coniferous and broad-leaved trees and is widespread in Asia and Europe [6]. It belongs to the Polyporaceae, traditionally categorized as reishi fungus in Far-Eastern Asia. The dry fruiting bodies of different *Fomitopsis* species have been used in folk medicine as haemostatics and anti-inflammation agents [7,8]. In addition, *F. pinicola* has been traditionally used as an anti-diabetic material in Korean folk medicine [9] and is considered a nontoxic mushroom in Europe [7]. The principle components of *F. pinicola*, such as triterpenes [10], steroids [7], and a heterogalactan [11] have been shown to exert medicinal effects, including antimicrobial action [7] and the inhibition of cyclooxygenase activity [10]. However, to the best of our knowledge, no report is available on the effect of *F. pinicola* on cancers.

In this article, we used different fungi water or ethanol extracts, including *F. pinicola*, *Ganoderma sinense* (*G. sinense*), *Fomitopsis officinalis* (*F. officinalis*), *Polyporus melanopus* (*P. melanopus*), *Taiwanofungus camphorates* (*T. camphorates*) to evaluate the corresponding anti-cancer activities in different cancer cell lines. We also clarified the possible mechanisms of *F. pinicola* in anti-cancer activity *in vivo* and *in vitro*.

## 2. Results and Discussion

### 2.1. Different Fungi Extracts Exhibit Anti-Tumor Activities in Different Cancer Cells

The number of new cancer cases each year is projected to rise worldwide by about 70% by 2030 due to demographic changes alone, with the largest increases in the lower-income countries. Lung, liver, colorectal and breast cancers are the most commonly diagnosed in males and females respectively, and these cancers also represent the most frequent types of cancer related deaths. Thus, medical advances in disease treatment are an issue at a later stage and should be made available globally [12]. In order to evaluate the anti-cancer activity of different fungal species, different cancer cells lines, including Homo sapiens hepatoma (HepG2), lung cancer (A549), colorectal cancer (HCT-116), breast cancer (MDA-MB-231) cells and *Mus musculus* sarcoma (S-180) cells were treated with same concentration at 50 µg/mL of the water or ethanol extracts of different fungi (Table 1). The tested fungal samples in the study, including *G. sinense* and *T. camphorates* have been reported to exert anti-cancer activities. *G. sinense* has an antiproliferation effect on tumors through both the apoptosis pathway and cell cycle arrest effects, and besides triterpenoids, some other compounds such as sterols and nucleosides may contribute to these activities [13]. Triterpenoids isolated from *T. camphorates* are considered to be potential anticancer agents [14]. Among the tested fungal samples, *F. officinalis*, *P. melanopus* and *F. pinicola* were not reported to have effects on cancer cells, so this is the first report indicating that *F. officinalis*, *P. melanopus*, and *F. pinicola* exert anti-cancer effects *in vitro*. Water extracts of the different fungi species exert more moderate anti-cancer activities than that of ethanol extracts. Some of the fungi species water extracts even shows no inhibitory effect on HepG2 and A549 cells. In contrast to water extracts, ethanol extracts of different fungal species show significant anti-cancer activities. In addition, among the various fungal ethanol extracts, *F. pinicola* ethanol extract exerts the strongest inhibitory effect on the tested cancer cell lines. To the best of our knowledge, this is the first report indicated that the ethanol extract of *F. pinicola* shows an anti-tumor activity, although it is extensively used in Asia and Europe as a non-toxic anti-diabetes fungus [9].

**Table 1:** Growth-inhibition effect of different reishi mushroom extracts on different cancer cell lines. Mouse sarcoma 180 cells (S-180), human hepatoma (HepG2), lung cancer (A549), colon cancer (HCT-116) and breast cancer (MDA-MB-231) cells were treated with different fungus water or ethanol extracts at a dose of 50 µg/mL for 24 h. The viability of the cells was determined by MTT assay. Data were presented as mean ± S.E.M, and obtained from three independent experiments with four replicates for each experiment. * *p* < 0.05, ** *p* < 0.01 and *** *p* < 0.001 compared with the cell viability of control group which was set as 100%.

	S-180	HepG2	A549	HCT-116	MDA-MB-231
*F. pinicola*					
Water extract	78.9 ± 5.6 *	96.6 ± 5.8	97.0 ± 2.4	62.5 ± 8.5 **	60.1 ± 7.2 **
EtOH extract	17.2 ± 3.4 ***	28.7 ± 7.5 ***	7.1 ± 3.2 ***	12.1 ± 2.9 ***	34.1 ± 5.1 ***
*G. sinense*					
Water extract	47.7 ± 3.5 **	94.6 ± 3.5	96.9 ± 2.3	72.1 ± 5.7 *	61.3 ± 6.9 **
EtOH extract	31.2 ± 10.2 ***	41.2 ± 10.2 **	80.3 ± 7.6 *	14.7 ± 2.1 ***	28.5 ± 4.8 ***
*F. officinalis*					
Water extract	64.0 ± 4.9 **	98.9 ± 0.9	97.9 ± 1.4	20.6 ± 6.2 ***	60.8 ± 8.8 **
EtOH extract	34.1 ± 9.1 **	31.1 ± 5.1 **	62.0 ± 4.4 *	15.7 ± 4.0 ***	28.8 ± 1.1 ***
*P. melanopus*					
Water extract	61.7 ± 8.1 **	99.9 ± 0.1	97.7 ± 0.9	48.4 ± 4.6 **	73.6 ± 4.9 *
EtOH extract	33.3 ± 7.7 ***	31.5 ± 2.7 **	82.5 ± 7.5 *	16.0 ± 3.3 ***	28.7 ± 5.8 ***
*T. camphoratus*					
Water extract	98.2 ± 5.6	44.8 ± 5.6**	94.6 ± 2.5	72.5 ± 6.7 *	51.1 ± 4.2 **
EtOH extract	48.5 ± 3.2 **	36.2 ± 6.2 **	9.8 ± 2.4 ***	12.9 ± 1.9 ***	29.1 ± 3.6 ***

### 2.2. Ethanol Extract of F. pinicola Significantly Decreases S-180 Cell Viability

**Figure 1:**
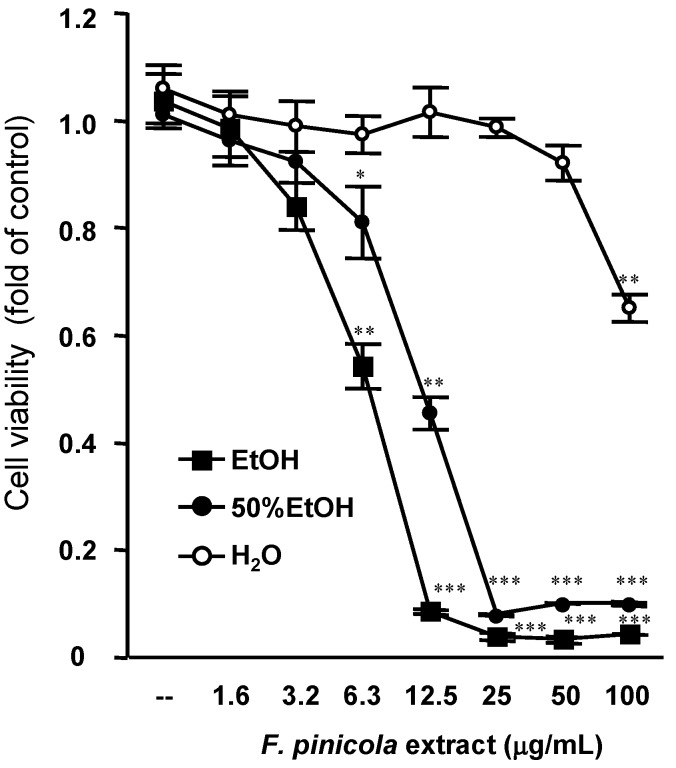
Anti-tumor effect of different *F. pinicola* extracts on S-180 cells. Sarcoma S-180 cells were treated with different *F. pinicola* extracts at indicated doses for 24 h. Cells were then incubated with 0.2 mg/mL MTT in culture medium for 4 h. MTT reagent was discarded and DMSO was added. The absorbance was measured at the wavelength of 570nm.The data is expressed as means±S.E.M. and obtained from three independent experiments with four replicates for each experiment. * *p* < 0.05, ** *p* < 0.01 and *** *p* < 0.001 compared with the control group.

**Figure 2:**
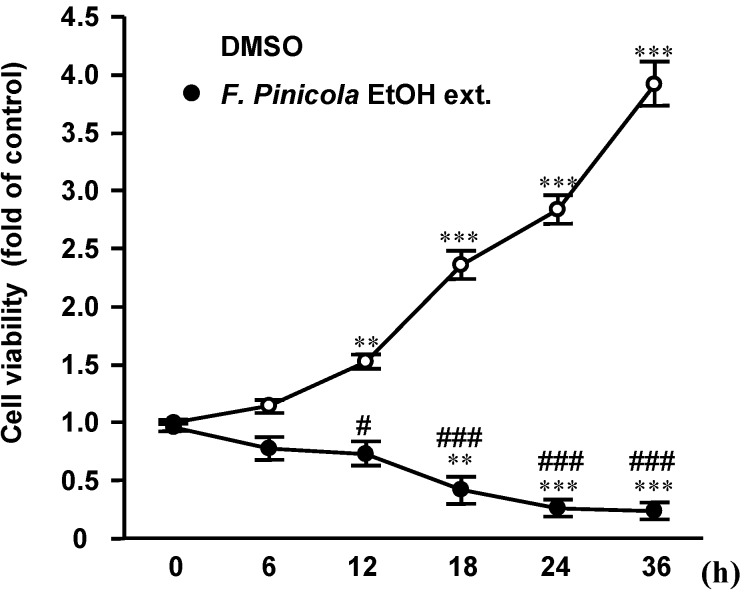
Ethanol extract of *F. pinicola* inhibits S-180 cells proliferation in a time-dependent manner. Sarcoma S-180 cells were treated with *F. pinicola* ethanol extract at a dose of 12.5 µg/mL for various durations as indicated. Cells were then incubated with 0.2 mg/mL MTT in culture medium for 4 h. MTT reagent was discarded and DMSO was added. The absorbance was measured at the wavelength of 570 nm.The data is expressed as means ± S.E.M and obtained from three independent experiments with four replicates for each experiment. * p < 0.05, ** p < 0.01 and *** p < 0.001 compared with the control group. #* p *< 0.05 and ###* p *< 0.001 compared with the *F. pinicola* ethanol extract-treated group.

We then investigated the half maximal inhibitory concentration (IC_50_) values of different *F. pinicola* extracts on S-180 cells (Figure 1). Ethanol extract of *F. pinicola* shows stronger inhibitory effect on cancer cells (IC_50_ = 7.4 µg/mL) than a 50% ethanol extract (IC_50_ = 12.2 µg/mL), whereas the inhibitory effect of water extract was weaker than that of the ethanol extracts (IC_50_ > 100 µg/mL). This result implies that the main components that contribute to the anti-tumor effects of *F. pinicola* may be hydrophobic. In addition, we found that *F. pinicola* ethanol extract inhibited S-180 cell growth at 12 h and significant cell death was observed at 24 h after treatment, implying the mediation of programmed cell death (Figure 2). The anticancer activities of fungi are mainly linked to the modulation of the immune system by branched polysaccharides (glucans), glycoproteins or peptide/protein-bound polysaccharides [15,16]. Moreover, mushrooms contain minerals, vitamins (e.g., thiamin, riboflavin, ascorbic acid, and vitamin D), amino acids, and other organic compounds [17]. Some of these natural mushroom compounds have demonstrated specific activity against aberrantly activated signaling pathways in cancer cells and were able to modulate specific molecular targets in the cell function including cell proliferation, cell survival and angiogenesis [18]. *F. pinicola* has been studied over the last years and a number of triterpenic compounds have been isolated [10,19], many of them possessing antimicrobial and anti-inflammatory activities. In this study, we found that *F. pinicola* ethanol extract also exerts an effect on anti-tumor.

### 2.3. Ethanol Extract of F. pinicola Induces Cell Apoptosis in Cancer Cells

**Figure 3:**
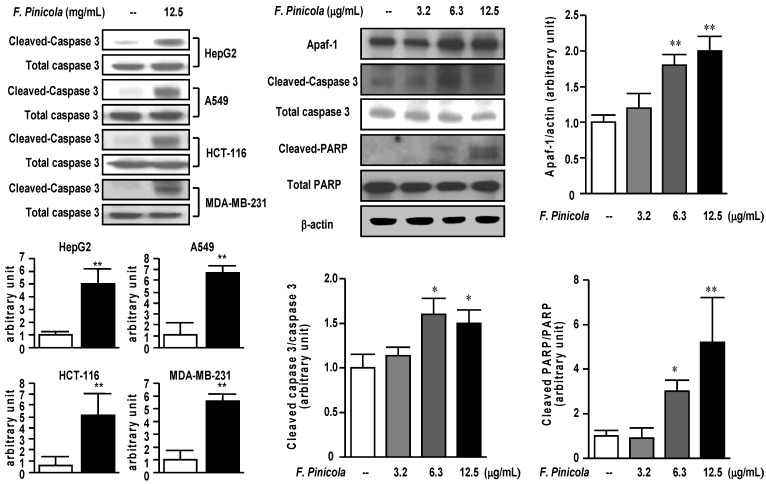
Ethanol extract of *F. pinicola* induced apoptosis to exert anti-tumor effect in cancer cells. Different human cancer cell lines as indicated were treated with 12.5 µg/mL *F. pinicola* ethanol extract for 18 h. The cleaved-caspase 3 levels were detected by western blots (**left** panel). Mouse sarcoma S-180 cells were treated with 12.5 µg/mL *F. pinicola* ethanol extract for 18 h. The apoptosis-related protein expressions were detected by Western blots (**middle** and **right** panels). * *p* < 0.05, and ** *p* < 0.01 compared with the control group.

We further investigated the possible mechanism of the inhibitory effect on cancer cells for *F. pinicola* ethanol extracts (Figure 3). Treatment of *F. pinicola* ethanol extract increased the expression of cleaved-caspase 3 in different cancer cell lines, implying the induction of cell apoptosis. In addition, we found the increased expression of apoptosis-related proteins, such as Apaf, cleaved-caspase 3 and PARP. Treatment of S-180 cells with *F. pinicola* ethanol extract significantly and dose-dependently increased the expression of Apaf, cleaved caspase 3 and PARP (Figure 3), implying the* F. pinicola* ethanol extracts induces cell apoptosis.

### 2.4. Anti-Tumor Activity of F. pinicola Ethanol Extract In Vivo

**Figure 4:**
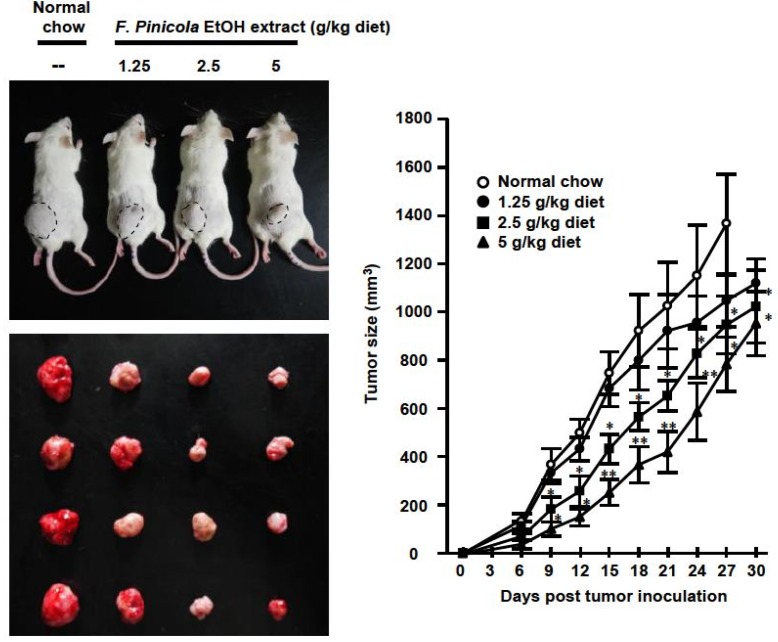
Administration of *F. pinicola* ethanol extract in S-180 tumor-bearing mice decreased tumor size and prolonged the survival time of the mice. Mice were pre-treated with a diet supplemented with different doses of *F. pinicola* ethanol extract as indicated for 3 days. Solid-type sarcoma S-180 was subcutaneously injected in mice on day 0. The indicated amounts of *F. pinicola* ethanol extract were supplied for 27 consecutive days. The tumor volume was determined by direct measurement with calipers and calculated every 3-day. Results are expressed as means ± S.E.M. *n* = 10. * *p* < 0.05 and ** *p* < 0.01 compared with normal chow group.

**Figure 5:**
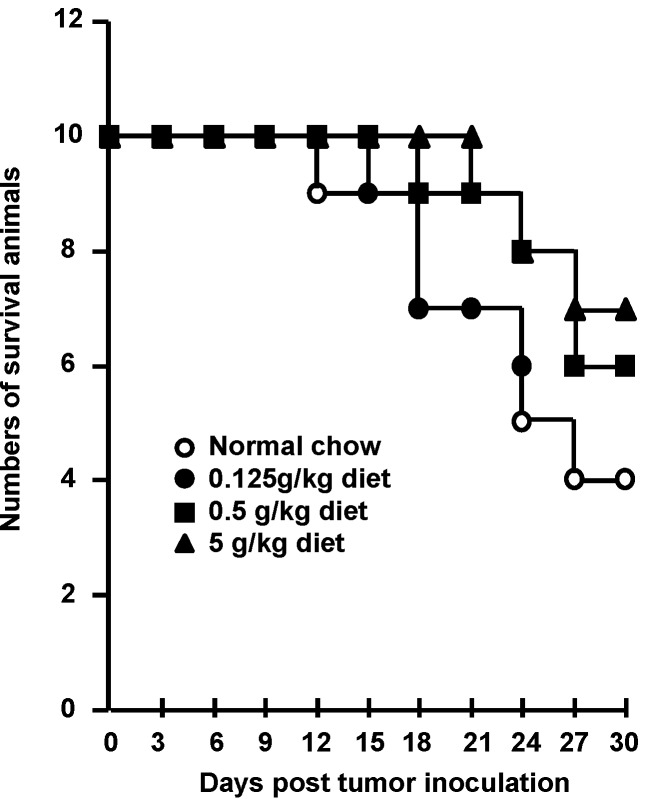
Administration of *F. pinicola* ethanol extract in S-180 tumor-bearing mice prolonged the survival time of the mice. Mice were pre-treated with a diet supplemented with different doses of *F. pinicola* ethanol extract as indicated for 3 days. Solid-type sarcoma 180 was subcutaneously injected in mice on day 0. The indicated amounts of *F. pinicola* ethanol extract were supplied for 27 consecutive days. Survival times of the S-180 tumor-beaing mice were recorded after after tumor inoculation. Results are expressed as the numbers of survival animals, *n* = 10.

To clarify the usefulness of *F. pinicola* ethanol extract as an alternative medicine for cancer therapy, mice were mice fed a diet containing 0.5% *F. pinicola* ethanol extract. As shown in Figure 4 this significantly inhibited 46% tumor growth without causing sudden death (data not shown). There was no marked weight loss in these mice after the high dose treatment of *F. pinicola* ethanol extract (data not shown). In addition, administration of *F. pinicola* ethanol extract prolonged the survival time of S-180 cell-bearing mice (Figure 5). It was reported that the constituents of *F. pinicola* regulate hyperglycemia via either increased insulin secretion during recovery or the prevention of streptozotocin-induced pancreatic damage and there is no evidence of toxicity in *F. pinicola* extract-treated rats [9]. In this study, no significant toxicity was observed in *F. pinicola* extract-treated mice, consistent with the previous study [9]. Therefore, ethanol extract of* F. pinicola* may be a new alternative medicine for the treatment of cancer.

### 2.5. F. pinicola Ethanol Extract Synergistically Enhances Anti-Tumor Activity of Cisplatin in S-180 Mouse Sarcoma Cells

**Figure 6:**
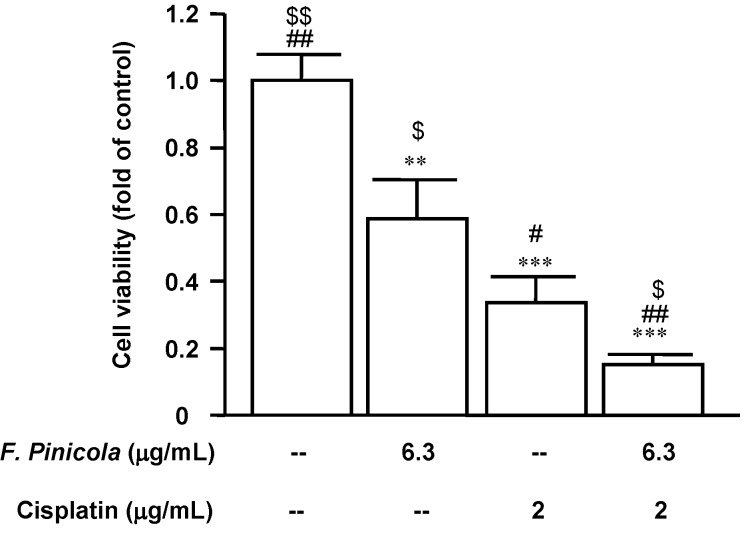
*F. pinicola* ethanol extract enhances the anti-tumor activity of cisplatin. Sarcoma S-180 cells were treated with *F. pinicola* ethanol extract, cisplatin or *F. pinicola* ethanol extract plus cisplatin at indicated doses for 24 h. Cells were then incubated with 0.2 mg/mL MTT in culture medium for 4 h. MTT reagent was discarded and DMSO was added. The absorbance was measured at the wavelength of 570 nm.The data was obtained from three independent experiments with four replicates for each experiment. ** *p* < 0.01 and *** * p* < 0.001 compared with the control group. ^#^* p* < 0.01 and ^##^* p* < 0.001 compared with *F. pinicola* ethanol extract-treated alone group. ^$^* p* < 0.05 and ^$$^* p* < 0.01 compared with cispltin-treated alone group.

In order to develop new therapeutic options with high efficacy and low toxicity, we tested by MTT assay the effectiveness of combining a conventional antineoplastic drug, cisplatin, with *F. pinicola* ethanol extract in the treatment of S-180 mouse sarcoma cells. We found that the use of *F. pinicola* ethanol extract as monotherapy reduced the viability of the S-180 cell line. Combining *F. pinicola* ethanol extract with cisplatin produced a synergistic effect and was more effective than was the use of cisplatin as monotherapy (Figure 6). Taken together, these results indicate that *F. pinicola* ethanol extract could be a potential alternative adjuvant anti-tumour medicine representing a promising approach to the treatment of some cancers in the future.

## 3. Experimental

### 3.1. Plant Material and Extraction

Different fungal fruit bodies were purchased from the Agricultural Production and Marketing Groups of the Hualien District Agricultural Research and Extension Station (Ji-An Country, Hualien County, Taiwan) in September 2011. The identification of the fungi was confirmed by macroscopic and microscopic examinations, as well as thin-layer chromatography and HPLC methods. The fungal fruit bodies were dried under shade at 25 °C. The dried samples (1 kg) were cut into small pieces, immersed, and extracted three times with ethanol, 50% ethanol or distilled water (10 L × 3) for 24 h at room temperature. After filtration, the solvent was removed by distillation under reduced pressure and the remaining solution was lyophilized to obtain a brown powder, and the samples were stored at 4 °C.

### 3.2. Cell Cultures

The cancer cell lines, HepG2 and S-180 cell lines were purchased from the Bioresource Collection and Research Center (Food Industry Research and Development Institute, Hsinchu, Taiwan). HCT-116 and MDA-MB-231 are gifts from Prof. M.D. Lai, and A549 is from Prof. C.L. Wu (Department of Biochemistry and Molecular Biology, College of Medicine, National Cheng Kung University, Tainan, Taiwan). Cells were maintained in Dulbecco’s modified Eagle medium (Hyclone, South Logan, UT, USA) supplemented with 10% heat-inactivated fetal bovine serum. Cell viability is determined by trypan blue staining and calculated as the number of viable cells divided by the total number of cells within the grids on the hemacytometer. If cells take up trypan blue, they are considered non-viable. % Viable cells = [1.00 − (Number of blue cells/Number of total cells)] × 100%. Cell viability should be at least 95% for healthy log-phase cultures.

### 3.3. MTT Assay

Cells (5 × 10^4^ cells per well) were seeded in a 96-well flat-bottom culture plate. After the additional treatments for indicated intervals, 100 µL of 0.2 mg/mL 3-(4,5-methylthiazol-2-yl)-2,5-diphenyl-tetrazolium bromide (MTT, USB Corporation, Cleveland, OH, USA) was added per each well, and cells were incubated for four hours at 37 °C. After incubation, the MTT reagent was discarded, and 100 µL of DMSO was then added. The experiment was performed at room temperature for 20 min. The absorbance was then measured with Multiskan GO spectrophotometer (Thermo Scientific, Waltham, MA, USA) at the wavelength of 570 nm. The viability of control group was set as 100% and the cell viability of each tested groups was calculated by the following formula: Cell viability (%) = (OD_treated_/OD_control_) × 100%.

### 3.4. Western Blotting

The cells were harvested at the indicated times and lysed with a buffer containing 1% Triton X-100, 50 mM of Tris (pH 7.5), 10 mM of EDTA, 0.02% NaN3, and a protease inhibitor cocktail (Sigma-Aldrich, St. Louis, MO, USA). The protein concentration was determined by BCA assay kit (Pierce Biotechnology, Rockford, IL, USA). Protein lysates (50 µg) were separated using 10% SDS-polyacrylamide gel electrophoresis and transferred to a polyvinylidene difluoride membrane (Millipore, Billerica, MA, USA). The membrane was blocked at room temperature for 1 h in TBS-T (10 mM Tris, 150 mM NaCl, and 0.05% Tween 20, [pH 7.6]), containing 10% skim milk, and probed with 1:1,000 primary antibodies, such as Apaf, caspase 3, PARP (Cell Signaling Technology, Beverly, MA, USA) and actin (Santa Cruz Biotechnology inc, Santa Cruz, CA, USA) at 4 °C overnight. Subsequently, the blots were washed with TBS-T and incubated with a 1:5,000 dilution of horseradish peroxidase-conjugated secondary antibodies at room temperature for 1 h. The protein bands were visualized using Immobilon™ (Millipore). Actin was used as the internal control. The relative signal intensity was quantified using ImageJ software from W. Rasband (National Institutes of Health, Bethesda, MD, USA).

### 3.5. S-180 Bearing Animal Model

Eight-week-old BALB/c male mice were purchased from the Animal Center of National Cheng Kung University Medical College. Mice were housed in a temperature (25 ± 1 °C) and humidity (60% ± 5%) controlled room and kept on a 12:12 light-dark cycle (light on at 0600). The animal procedures were performed according to the Guide for the Care and Use of Laboratory Animals of the National Institutes of Health, as well as the guidelines of the Animal Welfare Act. The mice were fed with a chow diet supplied with 1.25, 2.5, and 5 g/kg diet *F. pinicola* ethanol extract for 3 days, and then S-180 tumor cells (10^7^ cells/200 μL) were implanted subcutaneously into the left hind groin of the experimental mice followed by a previous study [20].

### 3.6. Statistical Analysis

Data are expressed as means ± S.E.M. Student’s *t*-test was used to determine the source of significant differences where appropriate. Significance was declared when the *p* value was less than 0.05.

## 4. Conclusions

Ethanol extracts of different fungal species show significant inhibitory effects on cancer cells as compared with the water extracts. In addition, among the different fungi species, *F. pinicola* ethanol extract induces cancer cell apoptosis to display a strong anti-cancer activity* in vitro* and* in vivo*. *F. pinicola* ethanol extract may serve as a potential complementary and alternative medicine to treat patients suffering from cancer.

## References

[1] Zheng R., Jie S., Hanchuan D., Moucheng W. (2005). Characterization and immunomodulating activities of polysaccharide from Lentinus edodes. Int. Immunopharmacol..

[2] Yuen J.W., Gohel M.D. (2005). Anticancer effects of Ganoderma lucidum: A review of scientific evidence. Nutr. Cancer.

[3] Kim H.J., Chang W.K., Kim M.K., Lee S.S., Choi B.Y. (2002). Dietary factors and gastric cancer in Korea: A case-control study. Int. J. Cancer.

[4] Hara M., Hanaoka T., Kobayashi M., Otani T., Adachi H.Y., Montani A., Natsukawa S., Shaura K., Koizumi Y., Kasuga Y. (2003). Cruciferous vegetables, mushrooms, and gastrointestinal cancer risks in a multicenter, hospital-based case-control study in Japan. Nutr. Cancer.

[5] Zhang M., Huang J., Xie X., Holman C.D. (2009). Dietary intakes of mushrooms and green tea combine to reduce the risk of breast cancer in Chinese women. Int. J. Cancer.

[6] Rosecke J., Pietsch M., Konig W.A. (2000). Volatile constituents of wood-rotting basidiomycetes. Phytochemistry.

[7] Keller A.C., Maillard M.P., Hostettmann K. (1996). Antimicrobial steroids from the fungus Fomitopsis pinicola. Phytochemistry.

[8] Zjawiony J.K. (2004). Biologically active compounds from Aphyllophorales (polypore) fungi. J. Nat. Prod..

[9] Lee S.I., Kim J.S., Oh S.H., Park K.Y., Lee H.G., Kim S.D. (2008). Antihyperglycemic effect of Fomitopsis pinicola extracts in streptozotocin-induced diabetic rats. J. Med. Food.

[10] Yoshikawa K., Inoue M., Matsumoto Y., Sakakibara C., Miyataka H., Matsumoto H., Arihara S. (2005). Lanostane triterpenoids and triterpene glycosides from the fruit body of Fomitopsis pinicola and their inhibitory activity against COX-1 and COX-2. J. Nat. Prod..

[11] Usui T., Hosokawa S., Mizuno T., Suzuki T., Meguro H. (1981). Investigation of the heterogeneity of heterogalactan from the fruit bodies of Fomitopsis pinicola, by employing concanavalin A-Sepharose affinity chromatography. J. Biochem..

[12] Franceschi S., Wild C.P. (2013). Meeting the global demands of epidemiologic transition—The indispensable role of cancer prevention. Mol. Oncol..

[13] Liu Y.W., Gao J.L., Guan J., Qian Z.M., Feng K., Li S.P. (2009). Evaluation of antiproliferative activities and action mechanisms of extracts from two species of Ganoderma on tumor cell lines. J. Agric. Food Chem..

[14] Bishayee A., Ahmed S., Brankov N., Perloff M. (2011). Triterpenoids as potential agents for the chemoprevention and therapy of breast cancer. Front. Biosci..

[15] Wasser S.P. (2002). Medicinal mushrooms as a source of antitumor and immunomodulating polysaccharides. Appl. Microbiol. Biotechnol..

[16] Borchers A.T., Krishnamurthy A., Keen C.L., Meyers F.J., Gershwin M.E. (2008). The immunobiology of mushrooms. Exp. Biol. Med..

[17] Mattila P., Suonpaa K., Piironen V. (2000). Functional properties of edible mushrooms. Nutrition.

[18] Zaidman B.Z., Yassin M., Mahajna J., Wasser S.P. (2005). Medicinal mushroom modulators of molecular targets as cancer therapeutics. Appl. Microbiol. Biotechnol..

[19] Petrova A., Popov S., Gjosheva M., Bankova V. (2007). A new triterpenic alcohol from Fomitopsis pinicola. Nat. Prod. Res..

[20] Bezerra D.P., Castro F.O., Alves A.P., Pessoa C., Moraes M.O., Silveira E.R., Lima M.A., Elmiro F.J., Costa-Lotufo L.V. (2006). *In vivo* growth-inhibition of Sarcoma 180 by piplartine and piperine, two alkaloid amides from Piper. Braz. J. Med. Biol. Res..

